# Reduced Precision Underwrites Ego Dissolution and Therapeutic Outcomes Under Psychedelics

**DOI:** 10.3389/fnins.2022.827400

**Published:** 2022-03-17

**Authors:** Devon Stoliker, Gary F. Egan, Adeel Razi

**Affiliations:** ^1^Turner Institute for Brain and Mental Health, Monash University, Clayton, VIC, Australia; ^2^Monash Biomedical Imaging, Monash University, Clayton, VIC, Australia; ^3^Wellcome Centre for Human Neuroimaging, University College London (UCL), London, United Kingdom; ^4^CIFAR Azrieli Global Scholars Programs, Canadian Institute for Advanced Research (CIFAR), Toronto, ON, Canada

**Keywords:** precision, hierarchical predictive coding, psychedelics, ego dissolution, belief updating

## Abstract

Evidence suggests classic psychedelics reduce the precision of belief updating and enable access to a range of alternate hypotheses that underwrite how we make sense of the world. This process, in the higher cortices, has been postulated to explain the therapeutic efficacy of psychedelics for the treatment of internalizing disorders. We argue reduced precision also underpins change to consciousness, known as “*ego dissolution*,” and that alterations to consciousness and attention under psychedelics have a common mechanism of reduced precision of Bayesian belief updating. Evidence, connecting the role of serotonergic receptors to large-scale connectivity changes in the cortex, suggests the precision of Bayesian belief updating may be a mechanism to modify and investigate consciousness and attention.

## Introduction

Classic psychedelics, including psilocybin and lysergic diethylamide acid (LSD), at sufficient doses, can induce a range of subjective effects that include visual alterations (Studerus et al., 2012), altered perception of time (Wittmann et al., 2007) and shifts in attention and perspective that reframe the relationship of self with the world (Swanson, 2018). This latter phenomenon, known as *ego dissolution*, is suggested to be the fundamental feature of classic psychedelic psychoactive effects (Stoliker et al., 2021). Ego dissolution, or the shift in sense of self and dissolution of boundary between the self and world (Lebedev et al., 2015; Millière, 2017), has been validated as a measurable construct on the Ego Dissolution Inventory^1^ (Nour et al., 2016), with evidence suggesting that therapeutic outcomes are tied to its occurrence^2^ (Garcia-Romeu et al., 2014; Majic et al., 2015; Carhart-Harris and Goodwin, 2017; Roseman et al., 2018; Yaden and Griffiths, 2020). Ego dissolution and all subjective effects from classic psychedelics originate from the binding kinetics of the 5-HT2A receptor to psychedelic compounds (Quednow et al., 2012; Preller et al., 2019) with other 5-HT receptors and is suggested to mediate psychedelic experiences (Vollenweider and Preller, 2020). The 5-HT2A is a serotonergic receptor involved in attention, especially in the prediction of the cause of sensory impressions and the consequences of self-initiated actions (Picard and Friston, 2014).

The influence of psychedelics on serotonergic receptors are thought to underpin the veracity of beliefs by alterations to predictive processing (Carhart-Harris and Friston, 2019). Predictive processing describes the interplay between ascending neuronal signals—in the form of ascending prediction errors—and descending predictions of the cortical hierarchy that embody internal world models (Rao and Ballard, 1999; Friston, 2008). Synaptic communication between each layer of the cortical hierarchy and the layer immediately preceding it compares predictions and prediction errors to fulfill the core function of the brain: to reduce surprise (Friston and Kiebel, 2009; Friston, 2010; Clark, 2015). Surprise (surprisal) is described mathematically by the Free Energy Principle (FEP), as minimizing (variational) free energy (Friston et al., 2006; Friston and Kiebel, 2009). Ascending signals which contradict predictions generate prediction errors which are “surprising” and therefore garner attentional resources. Access to the appropriate level of the cortical processing hierarchy which hosts sufficient associative complexity is necessary to resolve the prediction error and thereby minimize surprise^3^ (Millidge et al., 2021). This can occur through predictive processes that guide attention to update predictions (Friston, 2003)^4^ or by initiation of actions on the world to align sensory evidence with predictions (Friston et al., 2009).^5^ Both strategies operate to minimize the difference (i.e., prediction error) between predictions and the sensory input.

The function of the cortical hierarchy to minimize surprise (or prediction errors)—according to the predictive coding paradigm—entails the maintenance of a generative model of the world which hosts beliefs about the causes and consequences of exogenous and endogenous sensations^6^ (Parr and Friston, 2018; Millidge et al., 2021). A cornerstone of the *generative* aspect of the model is the ability to update beliefs.^7^ Belief updating, known as Bayesian inference, represents a unifying principle in neural computation (Knill and Pouget, 2004). The nervous system forms a model of the cause and effect structure of the world (i.e., the causes of sensation and consequences of action) and according to Bayes’ rule reduces prediction errors by updating estimates of the likely causes of sensory information. These estimations are biased by prior beliefs derived by internal (generative) models of the causal structure of the world. Once a causal explanation of prediction errors is produced, prior beliefs are able to update, and form new posterior beliefs (Bennett, 2015). Belief updating is (here suggested as) the process of moment-to-moment experience witnessed as consciousness and influenced by attention.

Beyond the role of prediction of the nature of sensory signals, predictive processing also estimates a signal to noise ratio of prediction errors that modulate how prediction errors can be used to update predictions across the predictive hierarchy. This measure of certainty of sensory signals, known as *precision*, acts as a neural implementation of attention between layers of the cortical hierarchy—for example, in low levels of the hierarchy that precede conscious awareness (Millidge et al., 2021; Yon and Frith, 2021). The generative model encodes the causes of sensory states, and the confidence of predictions, in predictive processes which are more or less independent of high-level beliefs. For example, in the immediate (unconscious) inference about hidden states of the world through the construction of sensory impressions such as vision and audition.^8^ Information gained from these lower-level sensory impressions may form a prediction error and entail an adjustment of higher-level beliefs. For example, the belief there is no vehicle approaching while crossing a road may be adjusted by receiving updated sensory information of the speedy approach of a vehicle, demonstrating that predictive processing between ascending sensation and descending cognition (i.e., predictions) coordinate to update beliefs. When the prediction of a particular level in the hierarchy requires an update (i.e., experiences contradiction), the prediction error is attended and granted access to higher levels where prediction errors are resolved. In the above case, high level cognitive beliefs are updated, and action may be taken to minimize the error in the prior belief to avoid being struck by a moving vehicle. This mechanism requires that sensory prediction errors have a pathway facilitated through automated (sub-personal) attention to a higher processing level where conscious cognitive processes are able to minimize an error using top-down attentional resources. Predictive processing therefore suggests a means to examine and understand cognition—not an entirely new endeavor (Hohwy, 2020; Rorot, 2021). Attempts to bridge alternate methods of examining cognition, such as the analysis of large-scale resting state network connectivity, may aid the interpretation of predictive processes in consciousness and attention. Psychedelics present a unique opportunity to observe changes to large-scale connectivity in an altered state of consciousness that influences attention. We suggest precision—which corresponds to the modulation of prediction error signals underlying attention in predictive processing—may help unify multiscale connectivity changes and advance our understanding of consciousness and attention.

Precision—or the inverse of the variance of a signal (or noise)—is explored in this work as a bridge that connects belief updating across predictive processing with large-scale brain connectivity. Precision is thought to play a key role in predictive processes that encode predictions of sensory stimuli by encoding the intrinsic uncertainty of predictions that select channels of attention and their salience (Adams et al., 2013; Kanai et al., 2015; Millidge et al., 2021). Precision is useful in a dynamic environment by allowing a generative model of any organism to estimate a level of uncertainty about beliefs and their causal construction. This affords the opportunity to grant flexibility that enables adaptation to changing contexts. Precision can be modeled as a probabilistic range of prior alternate hypotheses about the causes of sensory information. Very confident beliefs represent high precision of neuronal signalling and suggest a lower probability that alternate hypotheses will be cognitively explored through attention and subsequently selected. In the instance of crossing the road, a very precise belief that there is no oncoming traffic may preclude attentional mechanisms from initiating an action to visually scanning the road before attempting to cross based on exogenous sensory evidence. However, more abstract beliefs related to endogenous sensory evidence and the generative model of the self and world are similarly able to undergo updating. For example, a prior belief about the honesty of a person may be updated by contradictory sensory information. Greater precision of beliefs at higher levels of the cortical processing hierarchy is indicative of an individual’s confidence in the accuracy of their generative model of the world.^9^ This confidence can modulate precision (and thus attentional resources) to the prediction errors at lower levels of the hierarchy (Yon and Frith, 2021). Consequently, a high level of belief in the honesty of, for example, one’s partner, may inhibit contrary sensory evidence from updating interpersonal or abstract (higher level) beliefs, which suggests a close association of precision to top-down attention that underlies the range of hypotheses and underwrites the updating of conscious beliefs.

**FIGURE 1:**
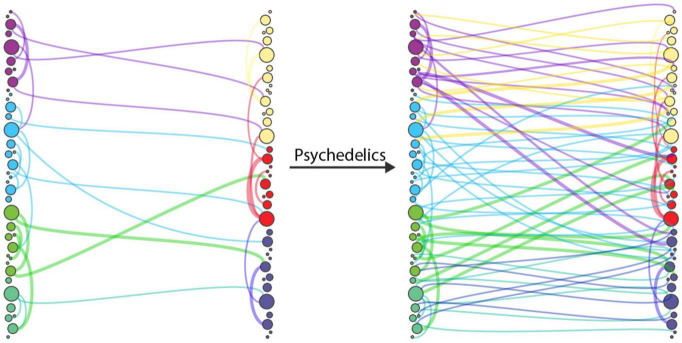
Illustration of desegregated connectivity under psilocybin, inspired by Petri et al. (2014). Change between cortical communities’ diversity of connectivity under psilocybin suggests a foundation of subjective effects (and facet of ego dissolution) that may be underwritten by reduced precision (i.e., confidence) of belief updating enabling connectivity to select from a wider range of hypotheses.

Despite precision being closely related to the self, it is significantly dependent on and modulated by neurotransmitter systems (Yon and Frith, 2021). Upper levels of the cortical hierarchy involved in consciousness and attention are abundant in serotonergic 5-HT2A receptors (Saulin et al., 2012; Carhart-Harris and Friston, 2019). The crucial role of serotonergic receptors in consciousness and attention is underscored by their function in the prediction of both the cause of sensory impressions and the consequences of self-initiated actions (Picard and Friston, 2014). Accordingly, agonism of the serotonergic 5-HT2A receptors by psychedelics is thought to reduce the precision of belief updating and reduce the “confidence” (or certainty) in the selection of any one channel of attention (Carhart-Harris and Friston, 2019). This has been postulated to afford a greater range of alternate hypotheses associated with the prior beliefs of a generative model (Carhart-Harris and Friston, 2019, see Figure 1). The effect of psychedelics on precision is supported by electrophysiological evidence that suggests a “flattening” of the free energy landscape^10^ (Bastos et al., 2012; Carhart-Harris and Friston, 2019), observations of increased entropy (Herzog et al., 2020) and theorized associations with increases in plasticity^11^ (Carhart-Harris, 2021; de Vos et al., 2021; Inserra et al., 2021).

### Ego Dissolution Underlies Therapeutic Benefits of Psychedelics

The previously described mechanisms of precision and predictive processing, as they apply to therapeutic modes of belief updating under psychedelics, are outlined in the 2019 work of Carhart-Harris and Friston (2019). They suggest the reduced precision of prior beliefs under psychedelics increases the range of hypotheses that can be entertained, and greater attentional resources allocated to alternate hypotheses as the product of reduced precision—with potentially therapeutic outcomes under proper guidance and supportive conditions. We extend this argument and suggest beliefs that undergird the self are updated in moment-to-moment experiences and that reduced precision of belief updating under psychedelics facilitates a change to consciousness (i.e., ego dissolution). Moreover, we suggest ego dissolution is a change in consciousness that reorients attention and enables therapeutic revisions of prior beliefs. This indicates the interplay between consciousness and attention are altered by classic psychedelics and underwritten by common changes to the precision of belief updating. The interplay between consciousness and attention is here generally hypothesized as *attention producing consciousness and consciousness focusing attention*. This formula follows a model of bottom-up sensory predictive processing, and crucial precision mechanisms selecting channels of attention, that manifest in higher levels of the cortical hierarchy as conscious impressions. In turn, these conscious impressions influence how top-down (higher) levels coordinate executive processes to focus attention. This theorization of the interplay is consistent with earlier descriptions of consciousness that direct top-down attention (Nani et al., 2019). However, notable delineation between top- down attention and consciousness indicate these as distinct processes (Koch and Tsuchiya, 2007). Under this premise, we suggest reduced precision under ego dissolution affect the top- down processes responsible for binding and making information accessible to planning parts of the brain, associated with the generative model of the self and world. We also recognize the role of reduced precision under psychedelics affecting bottom-up attention that contribute to the multimodal binding of the generative model. We demonstrate that alignment between predictive processing and larger scale connectivity changes in the brain may be dependent on reduced precision of belief updating, and describe reduced precision of belief updating as the trigger to alterations in consciousness and attention. We describe bottom-up and top-down attention processes that may uniquely contribute to ego dissolution (i.e., the change to conscious self-awareness and perception of boundary between the self and world), with a specific focus on hierarchical predictive processes and large scale brain connectivity.

The importance of *the self* and consciousness and attention in therapeutic outcomes and occurrences of ego dissolution inspire a bottom up and top down approach to understand the influence of precision of belief updating. The *bottom-up approach* recognizes consciousness as a constructive process of inference operating at the level of neural populations throughout the cortical hierarchy and that precision weighting influences the selection of attentional resources that integrate multimodal sensory connections (Millidge et al., 2021; Yon and Frith, 2021). This type of integration has been suggested to form the basis of phenomenal consciousness (Gallagher, 2000; Seth, 2013; Millière, 2017) as a description of how reality appears to us (Block, 2007; Nani et al., 2019). We suggest change to precision of bottom-up predictive processes may be important to psychedelic subjective effects, such as ego dissolution, and refer to large scale connectivity evidence that suggests the reduced filtering of the contents of consciousness under psychedelics is explained by filtration theory. Filtration theory describes the result of inhibition of filtering processes in the brain by the relaxation of constraints on perception, emotions, thought, and sense of self (Osmond, 1957; Marshall, 2005; Swanson, 2018). Change to bottom-up filtration under the control of selection processes of attention may have significant implications for multimodal integration of the conscious sense of embodiment. Psychedelics have the potential to help investigate how bottom-up changes to the precision of belief updating may be an important piece of the puzzle to understand changes of attention, consciousness, and self.

The *top-down approach* acknowledges the role of precision in high-level cognition associated with large-scale resting state networks (Yon and Frith, 2021). High levels of the cortical hierarchy integrate ascending multimodal signals with reference to the self and redirect attention based on goal directed salience (Menon, 2015). A focus on the upper most levels of the cortical hierarchy is pertinent to the exploration of psychedelics due to the abundant distribution of 5-HT2A receptors at this level (Saulin et al., 2012; Carhart-Harris and Friston, 2019). The receptor abundance is key as it may reduce the precision of predictions that have an inhibitory effect upon bottom up signals (Carhart-Harris and Friston, 2019). Self-related aspects of phenomenal consciousness, such as self-identity, can be explored through functional magnetic resonance imaging (fMRI) studies that have associated connectivity of networks and regions to self-related functions and identified connectivity alteration in psychedelic-induced ego dissolution. Interpretation of this evidence with reference to the mechanism of precision suggests precision of belief updating may underwrite psychedelic-induced ego dissolution and by extension, the therapeutic opportunities to redirect bottom-up and top-down attention in the ego dissolution state of consciousness. The exploration of precision under psychedelics focuses on the salience and default mode networks. The function of these networks in consciousness overlaps with the self (i.e., ego^12^) and serves as a primary example of reduced precision, or confidence, that underpins the subjective experience of ego dissolution. Evidence of their “disintegration” (reduced functional connectivity) under psychedelics may be explained by loss of precision leading to reduced belief updating of the model of self that produces occurrences of ego dissolution. However, firstly we turn our attention to the role of bottom-up salience detection and discuss its potential role in bottom-up precision of belief updating during experiences of ego dissolution.

### Bottom-Up Salience

Phenomenal selfhood, also referred to as the minimal or embodied self, describes the basic, pre-reflective, sense of self as being rooted in sensory motor processes (Blanke and Metzinger, 2009; Legrand and Ruby, 2009; Limanowski and Blankenburg, 2013; Apps and Tsakiris, 2014). Embodiment connects and separates the self from the world. It relies upon the proper integration of multisensory signals from visual, somatosensory, interoceptive, and vestibular feedback (Millière, 2017). The integration of multisensory signals through predictive processes describes the belief updating of sensory impressions and detection of salient features in the environment (Menon, 2015). Salience detection involves the fast, automatic, filtering of perceptual features that are infrequent or of biological importance, as detected along the ascending neural pathways of the predictive hierarchy (Knudsen, 2007). Change to the precision of bottom-up predictive processes of belief updating may have a significant effect on multimodal integration that manifests in phenomenal consciousness and influences the experience of embodiment. Freer interactions identified lower in the cortical hierarchy under psychedelics support this idea (Carhart-Harris et al., 2014a). Research applying dynamic causal modeling, which estimates effective connectivity between brain regions, suggest freer interactions may relate to reduced thalamic filtration of neural signals to the cortex under LSD. Recent research demonstrated increased effective connectivity from the thalamus to the posterior cingulate cortex under LSD (Preller et al., 2019) which may indicate the selection processes of attention that constrain the contents reaching consciousness become disinhibited by psychedelics (Carhart-Harris and Friston, 2019; Preller et al., 2019). Functional MRI observations of freer interactions are also identified in the medial temporal lobe and are associated to heightened responses to sensory stimuli under psychedelics (Carhart-Harris et al., 2014a,b; Kaelen et al., 2016; Carhart-Harris, 2018). These findings correspond with the access of bottom-up prediction errors to higher levels of the cortical processing hierarchy that suggest reduced constraints (i.e., filtration processes) upon consciousness may contribute to experiences of ego dissolution. These changes to the integration of sensory impressions may therefore be underwritten by reduced precision of belief updating.

### Top-Down Salience

The automatic detection of salience from incoming sensory impressions is complemented by a secondary, higher-level, top-down system that similarly scans for salience. This brain system, known as the salience network, is context dependent and directs the focus of attention toward self-relevant, engaging, or rewarding stimuli (Seeley et al., 2007; Menon, 2015). It also serves to facilitate reaction and access to attention and working memory (Sridharan et al., 2008). Moreover, the salience network serves a key function coordinating attention between internal and external stimuli, which entail modes of consciousness (Sridharan et al., 2008). Reduced precision of top-down salience predictions may alter the ordinary goal directed focus of attention and have effects on the subjective structure of conscious experience. Reduced salience network activity has been detected in association with ego dissolution in an fMRI study of psilocybin (Lebedev et al., 2015). Furthermore, the diminution of anti-correlated activity between salience and default mode networks under the control of the salience network has been observed under psilocybin (Carhart-Harris et al., 2012). These findings suggest salience network connectivity changes coincide with psychedelic peak effects and ego dissolution. The diminution of anti-correlation between large-scale brain networks may be one of the mechanisms of ego dissolution by reduced precision of the salience network and allocation of attention to alternate large-scale networks. Reduced precision in the salience allocation of attentional resources intuitively aligns with the dissolution of boundaries reported in ego dissolution. Additionally, the influence of freer bottom-up ascending sensory impressions and reduced top down inhibition may compound to produce reduced constraints on consciousness. Reduced bottom-up and top-down precision underlying salience detection and the allocation of attention by the salience network are therefore hypothesized to jointly contribute to dissolving the sense of phenomenal self and the boundary between the internal and external world as reported in ego dissolution. We also argue that reduced precision of goal directed salience attention may contribute to a reprioritization of salience that is important to extending attention to alternate high level psychosocial hypotheses and contribute to the utility of therapeutic applications of psychedelics.

### Top-Down Narrative

Salience detection describes a foundation of phenomenal consciousness through ascending predictive processes whilst higher order salience functions of the salience network suggest the concept of top-down goal directed attention between internal and external modes of consciousness. The default mode network is also central to consciousness through its role in self-referential function and maintenance of passive awareness (Raichle et al., 2001; Buckner et al., 2008). Its expansion over the course of evolution (Van Essen and Dierker, 2007) suggests its core function to alert against predators during states of rest may have developed into sophisticated and uniquely human attributes, such as self-reflection (Qin et al., 2015) and a narrative sense of self. In Bayesian terms, the narrative self is the brain predicting its own contribution to sensory input by top-down inferences about the causes (i.e., latent or hidden states of the world) of sensory information (Metzinger, 2003; Friston, 2010; Clark, 2013). The narrative sense of self is semantically closer to the familiar conscious experience of possessing beliefs. The default mode network has also been suggested as the candidate neural substrate of the Freudian conceptualization of *ego* (Buckner et al., 2008; Carhart-Harris and Friston, 2010; Lebedev et al., 2015). Disintegration (deactivation) of the default mode network is one of the most frequently cited neural correlates of psychedelic-induced ego dissolution (Ruban and Kolodziej, 2018). Reduced functional connectivity of default mode network cardinal regions, such as the posterior cingulate cortex, are likely a key mechanism of disintegration and help to explain alterations of selfhood. The posterior cingulate cortex serves functions in self-reflection and its decreased oscillatory rhythms in the alpha range are observed under psilocybin (Muthukumaraswamy et al., 2013; Carhart-Harris et al., 2016). Notably, the range of oscillatory rhythms is thought to host beliefs in high level cortical areas (Carhart-Harris and Friston, 2010). Cortical oscillations suggest a link between 5-HT2A receptor agonism and observations of decreased functional connectivity of the default mode network under psychedelics. Translating the connectivity changes to psychological effects may be explained by reduced precision of belief updating. Such an interpretation carries intuitive appeal suggesting the basic function of region and network level connectivity underlies a sense of confidence that controls selection of top-down attention to beliefs among a range of alternatives that may be consciously entertained, and that consistency in predictions or precision of predictions, underlies ordinary states of consciousness. The role of oscillations that bind micro and macro scales of connectivity is further demonstrated by evidence that oscillatory synchrony is a means by which conscious experience is unified (Singer, 1999). Synchronization may be an expression of high precision. The influence of psychedelics to reduce precision and introduce greater cognitive flexibility aligns with the evidence that lagged synchronization and agonist activity of the 5-HT2A receptor underlies insightfulness: a facet of ego dissolution, with therapeutic potential (Kometer et al., 2015). This evidence may indicate a larger range of hypotheses that underlie belief updating and form the foundation of self and world that are entertained under psychedelics, and that the overlap between ego dissolution and psychedelic therapeutic potential may share a common origin to reduce precision of belief updating.

### Revisiting the Overlap Between Therapeutic Outcomes and Subjective Effects and Belief Updating

Change to salience and the default mode network may affect the foundation of consciousness through altered multimodal integration, direction of attention—and its influence on modes of consciousness—and changes to a stable sense of self-identity. These are considered important facets of ego dissolution that are underwritten by recurrent belief updating to maintain moment-to-moment conscious experience. Underlying large scale brain network connectivity changes, altered 5-HT2A receptor activity affects patterns of synaptic gain and influence the selection of channels attended and the deployment of attentional resources (Kanai et al., 2015; Carhart-Harris and Friston, 2019). Changes to attention contribute to inferences underlying beliefs. Reduced precision affords attention to alternate hypotheses underlying self-related beliefs, and this association is critically linked by the necessity of self-dissolving subjective effects to produce therapeutic outcomes (Roseman et al., 2018; Yaden and Griffiths, 2020). Although the association between psychedelic therapeutic effects and subjective effects remains debated (Ogawa et al., 1990), the evidence put forward here suggests reduced precision underlies both effects. This idea is further supported by the alignment of mental health disorders, to which psychedelics show efficacy in therapy and their association with internalized self-beliefs. These include addiction, anxiety, and depression (Johnson et al., 2014; Griffiths, 2015; Carhart-Harris et al., 2017, 2021). These disorders, grouped as internalizing conditions, involve maladaptive patterns of thought and behavior underwritten by self-related beliefs.

Personal beliefs, similar to sub-personal beliefs, may be underwritten by a change to the precision of parameters that constitute a generative model of identity. Although “belief updating” is more intuitively ascribed to beliefs one holds in association to oneself, one’s high- level generative model recurrently updates beliefs that constitute the nature of *the self*, from which self-held beliefs are hosted. The same predictive processes may then apply to both personal and sub-personal (automatic) forms of belief updating. Beliefs hosted by top-down high-level cortical networks such as the salience network and default mode network are highly distributed with 5-HT2A receptors (Carhart-Harris and Friston, 2019). The reduced efficiency of predictive processes can explain decreases in the efficiency of attention to minimize prediction errors (Hohwy, 2012), and ego dissolution as the reduced precision of recurrent belief updating underlying attention that manifests the self. Precision may therefore be a fundamental mechanism of the ego, ego dissolution, and by association, therapeutic effects pertaining to attention underwriting self-related beliefs.

### Desegregation and Precision

**FIGURE 2:**
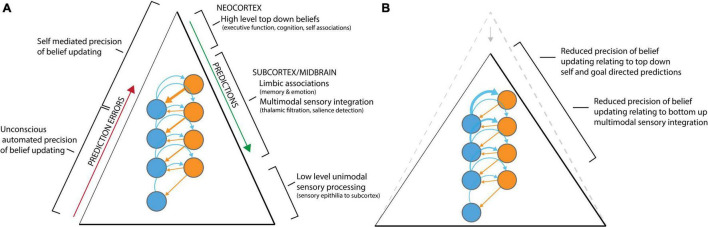
Simplified illustrative description of precision of belief updating along the cortical hierarchy. **(A)** Precision is represented along scales of the hierarchy and depicts the filtration of sensory impressions to cognition. Predictions and prediction errors can be seen to interact between each layer of the hierarchy, with stronger high-level predictions in the upper levels of the hierarchy. **(B)** Under psychedelics the precision of belief updating in high level predictions, involved in phenomenal consciousness and narrative self, is reduced. This occurs by belief updating opening alternate hypotheses that undergird neuronal populations “confidence” in the selection, and salience, of attention that integrate phenomenal consciousness and narrative self-identity. Reduced precision is represented by the reduced weight of predictions and increased influence of prediction errors in higher levels of the hierarchy where 5-HT2A receptors are abundant. The change to the precision of belief updating is hypothesized to result in ego dissolution. Elements of this model are inspired by Carhart-Harris and Friston (2019).

Figure 2 demonstrates the reduced “top heaviness” and flattening of the cortical hierarchy by reduced precision of beliefs, as reflected by *disintegration* of connectivity in resting state networks and regional activity. The disintegration of higher levels of the cortical processing hierarchy is observed alongside a secondary outcome of psychedelic-induced connectivity dynamics, which may be relevant to the experience of the dissolution of boundaries and altered multimodal sensory integration. This second effect, coined *desegregation*, is shown to be positively correlated with ego dissolution (Tagliazucchi et al., 2016). Desegregation describes the abundant deviation of connectivity from functional pathways—or decreased modularity in brain-networks and regions—and is an effect cited in reference to increased complexity—in an information theoretic sense—identified in the psychedelic state (Petri et al., 2014; Barnett et al., 2019, see Figure 1). Desegregation in cortical communities may relate the richness of psychedelic phenomenological experience which differs from other altered self-dissolving states of consciousness (Millière et al., 2018) that may relate to the complexity of a self-generative model (Rorot, 2021).

Increased complexity in the psychedelic state may relate to the important relationship between complexity and accuracy. Efficient communication requires a balance between accuracy (precision) and complexity (information entertained) (Bishop, 2006). The increased complexity of cortical connections in the psychedelic state may influence the generative models’ access to alternative hypotheses (prior beliefs) and sensory information (freer low- level interactions). Consequently, precision or confidence in the accuracy of any one prior belief may be reduced and stretch the imagination or range of alternative hypotheses within the generative model. This may dispose attention to unfamiliar hypotheses (and selection of connectivity pathways^13^) that underly the model of self and social exchanges with the world.

## Conclusion

Brain connectivity changes observed in psychedelic imaging research may be explained by the reduced precision of neuronal signaling in top-down and bottom-up hierarchical predictive processes. Under psychedelics, changes to precision are suggested to extend attentional resources to a broader range of hypotheses that operate to minimize prediction errors. Reduced efficiency of error minimization induced by psychedelics can offer the counter intuitive effect of breaking underlying maladaptive patterns of self-belief and offer attention to alternate hypotheses underlying construction of the world. In the light of evidence that psychedelics induce ego dissolution and its association to therapeutic outcomes, we argue that reduced precision alters belief updating which undergirds the phenomenal and narrative self that is experienced as ego dissolution. Psychedelic therapeutic effects may rely on the subjective change to consciousness, through a common change to attention processes underwritten by precision. Phenomenal consciousness and the sense of self are suggested as key facets of ego dissolution which are affected by the reduced confidence in reiterative belief updating under the action of psychedelics.

Precision is suggested to be ubiquitous in the nervous system. Precision bridges predictive processes across unconscious and conscious levels by acting as a measure of confidence. A natural extension to this is that precision of belief updating will then apply to numerous psychedelic effects such as visual hallucinations. Beyond psychedelics, precision may also apply to well-being, psychopathology, and by extension, basic facets of cortical function and perception. Precision may be one step in a complex sequence of interacting predictive process mechanisms. The necessity to comprehensively synthesize mechanisms of precision associated with the dynamics of consciousness and attention are acknowledged, as well as the limitation that precision alone may not provide an exhaustive account of predictive processing or connectivity mechanisms. Other facets of predictive processing, such as reference to the complexity of the generative model, interaction of precision across the hierarchy, and with the environment are equally important.^14^ However, precision does provide an intuitive way to interpret the translation of neural processes to consciousness. Conceptualizing precision as a key mechanism holds utility as an intuitive interpretation that explains the necessity of living organisms to recurrently update their beliefs to remain adaptive to an ever-changing environment, and more fundamentally, to remain conscious of their self and surroundings. This suggests the self is a constructive process of ongoing updating and that a useful way to understand predictive processing—both conscious and unconscious—is in terms of “beliefs.” Beliefs can be determined by precision, which represents a form of confidence, that at one end is psychological in nature, and at the other occurs in automatic sub-personal neuronal processes. Furthering our understanding of the relationship between the conscious and unconscious, and its relationship to attention, suggests a key utility of psychedelics for research in the cognitive sciences to experimentally probe predictive process theories of consciousness and attention^15^ and test their theoretical robustness.

## Data Availability Statement

The original contributions presented in the study are included in the article/supplementary material, further inquiries can be directed to the corresponding author.

## Author Contributions

DS: conceptualization and writing. GE and AR: writing—review and editing. All authors contributed to the article and approved the submitted version.

## Conflict of Interest

The authors declare that the research was conducted in the absence of any commercial or financial relationships that could be construed as a potential conflict of interest.

## Publisher’s Note

All claims expressed in this article are solely those of the authors and do not necessarily represent those of their affiliated organizations, or those of the publisher, the editors and the reviewers. Any product that may be evaluated in this article, or claim that may be made by its manufacturer, is not guaranteed or endorsed by the publisher.

## Footnotes

1 Validation of the ego dissolution inventory identifies the internal consistency of items measuring ego dissolution, their orthogonality to ego inflation, and correlation with unitive experience on the Mystical Experiences Questionnaire.

2 Ego dissolution is thought to be synonymous with peak experiences and mystical experiences, which are also terms used to describe the main existential subjective effect of classic psychedelics and have been related to therapeutic outcomes.

3 Prediction errors are the information in each layer that is not predicted by descending predictions.

4 Updating predictions can occur through immediate inference or updating the generative model of the world.

5 Predictions are referred to as synonymous with beliefs.

6 Both endogenous and exogenous signals are referred to as sensory signals/sensory impressions/sensory information.

7 A key factor of belief updating involves beliefs about hidden variables of the world (i.e., variables which cannot be directly observed and rely on inference).

8 This idea draws reference to Hermann Von Helmholtz’s idea that perception is unconscious inference and the notion of uncertainty being computed in sub-personal processes beneath the level of subjective awareness (Yon and Frith, 2021).

9 Precision is modulated by higher level cognition, such as cognitive biases, context and belief in the reliability of information. Exploration of these ideas is reviewed in Yon and Frith (2021).

10 The free energy landscape refers to the graph of the (high dimensional) free energy functional across the configuration state space of the system. This free energy landscape is then traversed to find the free energy minima, mathematically operationalized by the Bayesian variational free energy minimization (or the maximization of the log model evidence) algorithms. Visualizing the free energy landscape helps to explain how the network of local minima and transition states determines properties, such as cognitive flexibility, which signify transitions between free energy minima. The REBUS model is a description of the neural state-space optimization which theorizes that psychedelics flatten the shape (i.e., curvature) of free energy landscape hence it is easier to transit between cognitive (and brain) states resulting in higher cognitive flexibility. The REBUS model associates to the updating of posterior beliefs, and by extension the range of hypotheses, or precision, underlying belief updating.

11 Neuroplasticity in psychedelic research is hypothesized and supported by early research, that suggest neuroplasticity primarily mediation by the modulation of Brain-Derived Neurotrophic Factor (BDNF). Plasticity mechanisms on a molecular, neuronal, synaptic, and dendritic level are also under investigation.

12 *Narrative self*, which is closely related to the Freudian concept of ego, is identified in relation to the default mode network (Buckner et al., 2008; Carhart-Harris and Friston, 2010; Lebedev et al., 2015).

13 If we imagined the distribution of variance as a sort of compass, we might hypothesize some association between reduced precision selecting alternative hypotheses and the desegregation of large-scale connectivity pathways.

14 Complexity is suggested as an important feature alongside precision. Interested readers are directed to Rorot (2021). For review of precision and the Bayesian brain (see Yon and Frith, 2021).

15 These theories are not explored in this review. Interested readers are directed to Hohwy (2020) and Rorot (2021).

## Glossary

### Attention

Information that is relevant and selectively attended.

### Brain-Derived Neurotrophic Factor (BDNF)

BDNF (abrineurin) is a protein found in the brain that signals cells to survive, differentiate and grow and is also related to improved nerve transmission.

### Consciousness

Quality or state of subjective awareness.

### Desegregation

Increased global functional integration between cortical regions and decreased modularity between the different brain networks.

### Disintegration

Decreased functional connectivity between cortical regions composing large scale brain networks.

### Ego Dissolution

Subject effect of classic psychedelics involving the shift in sense of self and dissolving of subject-object boundaries (blurring self-boundaries).

### Generative Model

Generative models are models of how observations are generated by events outside of the brain that cannot be known directly. Mathematically, a model for randomly generating observations, typically given some (hidden) model parameters.

### Model Evidence

Model evidence—mathematically known as the marginal likelihood—forms the sole basis of Bayesian model selection, i.e., the ranking of competing models in the light of the data, with the highest evidence identifying the best model.

### Free Energy

Usually due to the high dimensionality of the problem, model evidence is intractable to calculate hence a lower bound is computed known as free energy. Simply, free energy is the lower bound on (log) model evidence used for model selection; higher free energy indicates better fit of data.

### Free Energy Landscape

The free energy landscape refers to the graph of the (high dimensional) free energy functional across the configuration state space of the system.

### Precision

The inverse of the variance (or uncertainty) associated with an observed signal.

### Prediction Error

The bottom-up signal which is defined by the difference between the top-down predictions (generated by the internal generative model) and the incoming sensory signals.

### Predictive Processing/Coding

A theory that brain function generates and updates a mental model of the environment to reduce prediction errors (or free energy).
